# Luminance Contrast Shifts Dominance Balance between ON and OFF Pathways in Human Vision

**DOI:** 10.1523/JNEUROSCI.1672-22.2022

**Published:** 2023-02-08

**Authors:** Hamed Rahimi-Nasrabadi, Veronica Moore-Stoll, Jia Tan, Stephen Dellostritto, JianZhong Jin, Mitchell W. Dul, Jose-Manuel Alonso

**Affiliations:** Department of Biological and Visual Sciences, College of Optometry, State University of New York, New York, New York 10036

**Keywords:** area V1, glaucoma, LGN, receptive field, thalamus, visual cortex

## Abstract

Human vision processes light and dark stimuli in visual scenes with separate ON and OFF neuronal pathways. In nature, stimuli lighter or darker than their local surround have different spatial properties and contrast distributions (Ratliff et al., 2010; Cooper and Norcia, 2015; Rahimi-Nasrabadi et al., 2021). Similarly, in human vision, we show that luminance contrast affects the perception of lights and darks differently. At high contrast, human subjects of both sexes locate dark stimuli faster and more accurately than light stimuli, which is consistent with a visual system dominated by the OFF pathway. However, at low contrast, they locate light stimuli faster and more accurately than dark stimuli, which is consistent with a visual system dominated by the ON pathway. Luminance contrast was strongly correlated with multiple ON/OFF dominance ratios estimated from light/dark ratios of performance errors, missed targets, or reaction times (RTs). All correlations could be demonstrated at multiple eccentricities of the central visual field with an ON-OFF perimetry test implemented in a head-mounted visual display. We conclude that high-contrast stimuli are processed faster and more accurately by OFF pathways than ON pathways. However, the OFF dominance shifts toward ON dominance when stimulus contrast decreases, as expected from the higher-contrast sensitivity of ON cortical pathways (Kremkow et al., 2014; Rahimi-Nasrabadi et al., 2021). The results highlight the importance of contrast polarity in visual field measurements and predict a loss of low-contrast vision in humans with ON pathway deficits, as demonstrated in animal models (Sarnaik et al., 2014).

**SIGNIFICANCE STATEMENT** ON and OFF retino-thalamo-cortical pathways respond differently to luminance contrast. In both animal models and humans, low contrasts drive stronger responses from ON pathways, whereas high contrasts drive stronger responses from OFF pathways. We demonstrate that these ON-OFF pathway differences have a correlate in human vision. At low contrast, humans locate light targets faster and more accurately than dark targets but, as contrast increases, dark targets become more visible than light targets. We also demonstrate that contrast is strongly correlated with multiple light/dark ratios of visual performance in central vision. These results provide a link between neuronal physiology and human vision while emphasizing the importance of stimulus polarity in measurements of visual fields and contrast sensitivity.

## Introduction

Cameras capture a visual image by transforming light intensity into a brightness–darkness scale. Because the wide luminance range of outdoor scenes frequently exceeds the camera luminance range, the captured images can lose visual detail by underexposing the dark features and/or overexposing the light features of the scene. Photographers address this problem by reducing the image exposure time and accepting a loss of visual detail in the dark features. Animal visual systems address this problem differently. They capture different images for light stimuli (Ls) and dark stimuli (Ds) with separate ON and OFF visual pathways that have different spatiotemporal contrast (C) properties closely matched to the statistics of light and dark stimuli in nature (Zemon et al., 1988; Chubb and Nam, 2000; Chubb et al., 2004; Ratliff et al., 2010; Jin et al., 2011a,b; Komban et al., 2014; Kremkow et al., 2014; Cooper and Norcia, 2015; Rekauzke et al., 2016; Pons et al., 2017; Jimenez et al., 2018; Ravi et al., 2018; Jansen et al., 2019; Norcia et al., 2020; Rahimi-Nasrabadi et al., 2021). Because dark stimuli are more informative than light stimuli in brightly illuminated worlds (Ratliff et al., 2010; Cooper and Norcia, 2015; Rahimi-Nasrabadi et al., 2021), the visual brains of carnivores and primates devote more neuronal resources to process information captured by OFF pathways than by ON pathways (Jin et al., 2008, 2011a; Yeh et al., 2009; Xing et al., 2010, 2014; Wang et al., 2015; Taylor et al., 2018; Oliveira Ferreira de Souza and Casanova, 2019), and can resolve greater visual detail for dark than light stimuli (Kremkow et al., 2014; Sato et al., 2016; Pons et al., 2017).

ON and OFF visual pathways are also differently affected by retinal degeneration in diseases such as glaucoma (Greenstein et al., 1998; Della Santina et al., 2013; El-Danaf and Huberman, 2015; Zhao et al., 2015; Ou et al., 2016; Sabharwal et al., 2017). The loss of vision resulting from retinal degeneration has been traditionally measured with light stimuli in visual field perimetry, a test that evaluates ON pathway but not OFF pathway function because brief light stimuli drive stronger transient responses from ON pathways than OFF pathways (Mazade et al., 2019). If ON and OFF pathways were equally driven by light stimuli, one stimulus polarity should be sufficient to evaluate both. However, there is increasingly stronger evidence that ON and OFF pathways have different functional properties (Chichilnisky and Kalmar, 2002; Zaghloul et al., 2003; Jin et al., 2008, 2011a,b; Yeh et al., 2009; Xing et al., 2010; Onat et al., 2011; Komban et al., 2014; Kremkow et al., 2014, 2016; Zurawel et al., 2014; Wang et al., 2015; Lee et al., 2016; Rekauzke et al., 2016; Pons et al., 2017; Luo-Li et al., 2018; Ravi et al., 2018; Taylor et al., 2018; Jansen et al., 2019; Mazade et al., 2019; Norcia et al., 2020; Rahimi-Nasrabadi et al., 2021), operate relatively independently throughout the visual system (Schiller et al., 1986; Dryja et al., 2005; Sarnaik et al., 2014), and are differently affected by retinal degeneration (Greenstein et al., 1998; Della Santina et al., 2013; El-Danaf and Huberman, 2015; Zhao et al., 2015; Ou et al., 2016; Sabharwal et al., 2017). Therefore, it is important to develop methods that evaluate ON and OFF pathway functions separately. Here, we introduce an ON-OFF perimetry test [Alonso JM, Dul MW, Nasrabadi HR (2021) U.S. Patent Application 17/217553, pending] that meets this requirement, and we use it to demonstrate that luminance contrast affects differently the visibility of light and dark stimuli. The contrast dependence of light/dark (LD) polarity dominance that we demonstrate is very robust and closely replicates differences in contrast sensitivity between ON and OFF pathways found in different species at different levels of the retino-thalamo-cortical pathway (Chichilnisky and Kalmar, 2002; Zaghloul et al., 2003; Kremkow et al., 2014; Pons et al., 2017; Ravi et al., 2018; Rahimi-Nasrabadi et al., 2021).

## Materials and Methods

### Human subjects.

We measured 34 eyes from 34 human subjects with ON-OFF visual field perimetry, a visual test that we developed to measure ON and OFF pathway function at different positions of the visual field. All subjects had normal best-corrected visual acuity (20/20) and spanned a wide range of ages from 23 to 78 years (average age, 36 ± 18 years; 18 females, 16 males). Since the best-corrected visual acuity was normal in both eyes, we did not have a strict criterion to select the eye to be tested. Most subjects (emmetropes and some myopes) were asked to select their preferred eye and, in other myopes, we selected the eye with higher myopia to increase the diversity of eye axial lengths included in the study. All experiments were approved by the Institutional Review Board at the State University of New York College of Optometry and followed the principles outlined in the Declaration of Helsinki.

### ON-OFF visual field perimetry.

Subjects sat comfortably while wearing a head-mounted visual display that presented stimuli in different parts of the visual field (VIVE Pro Eye, HTC; refresh rate, 90 Hz; maximum luminance, 112 cd/m^2^). The subjects responded to stimuli presented in the right hemifield by pushing a button with their right hand, and to stimuli presented in the left hemifield by pushing a button with their left hand. A stimulus was recorded as “missed target” if the subject did not push a button within 2 s following the stimulus presentation. All stimuli were generated with Unity virtual reality engine (version 2018) and presented in one of the two independent screens from the head-mounted visual display (the screen in front of the eye that was not stimulated was dark). Each display was located ∼2 cm in front of each eye, subtended around 100° × 100° of total visual angle (1440 × 1600 pixels/eye) and generated pixel intensities linearly related with luminance (slope, 1.02; intercept, 0.5 cd/m^2^; *R*^2^, 0.999) that spanned 256 different values from ∼1 to 112 cd/m^2^.

Subjects were instructed to fixate on a central dot while searching for peripheral stimuli displayed at random spatial locations in their visual field. The subjects had to keep their eyes within a circular radius of ±2.5°, and eye movements outside of this window aborted the stimulus trial (aborted stimulus trials were repeated at a later time in the visual test). The fixation window was relatively large to make the visual test accessible to a large variety of subjects with a wide range of ages and visual conditions. Peripheral stimuli were light or dark square targets with a size that varied as a function of visual eccentricity (E), from a minimum of 2.5°/side at 5° to 4°/side at 30°. The increase in stimulus size was implemented with a power-law function defined as *S* = 2.5 (*E*/5)^0.26^, where *S* is the stimulus size in degrees per side and *E* is the visual eccentricity in degrees (the exponent was adjusted to minimize the differences in reaction time across eccentricities for dark stimuli at 20% C (C20). All stimuli were superimposed on a noisy background made of 131,072 equidistant triangles with 0.5°/side that tiled a sphere centered around the observer and changed randomly every trial. The probability that the background triangles formed a false target was very small (*p* ≤ 0.5^25^) because the side of the square targets were five to eight times larger than the side of the background triangles (e.g., there is 0.5 probability that one triangle is dark, 0.5^2^ probability that two contiguous triangles are dark, and 0.5^25^ probability that 25 contiguous triangles are dark). Before starting, all subjects received instructions to properly adjust the headset position, interpupillary distance, and perform the ON-OFF perimetry test. They also made saccades to a target presented at five different locations in the visual display to calibrate eye position. The headset adjustments and eye-tracking calibration lasted <1 min and were followed by 30 s of practice trials and adaptation to the background luminance.

The settings of ON-OFF perimetry allow sampling different numbers, positions, and geometries of peripheral stimuli in the human visual field. For this study, we sampled four quadrants separated in temporal/nasal hemifields within the azimuth axis and upper/lower hemifields within the elevation axis. The nasal quadrants of the visual field were sampled with more stimulus positions (24) than the temporal quadrants (20) to allow future comparisons with glaucoma patients, who tend to have more extensive damage in the temporal retinas sampling the nasal visual fields. We also sampled the position of the blind spot in the temporal hemifield (optic disk in the retina) and its mirror position in the nasal hemifield to estimate the noise level in the measurements. If the measurement noise is low (e.g., at high stimulus contrast), the subjects signal the presence of all stimuli in the nasal field and none in the blind spot at the optic disk. However, if the measurement noise is high (e.g., at low stimulus contrasts), the number of errors increases across the entire visual field, as subjects try to guess the target location more often. The blind spot and its mirror position were sampled with a small stimulus size (2°) to increase the noise level sensitivity. In total, we sampled 90 different stimulus positions, 20 in each temporal quadrant, 24 in each nasal quadrant, 1 in the blind spot, and 1 in its mirror location within the nasal field (20 × 2 + 24 × 2 + 1 × 2 = 90). Each spatial position was sampled six times, three times with a dark stimulus and three times with a light stimulus. The blind spot and its mirror spatial location were sampled six additional times to maximize our estimates of noise. Therefore, we ran a total of 552 stimulus trials (90 × 6 + 2 × 6 = 552). In addition, we ran 27 “catch” trials with no stimuli to estimate the subject selection criteria (i.e., subjects using more conservative criteria signal “no stimuli” more frequently in catch trials than those guessing more often). Therefore, in total, the test included 579 trials (552 trials with stimuli + 27 trials with no stimuli = 579 trials). The stimulus contrast was the same in all trials. When the stimulus contrast was high, the subjects completed most stimulus trials within 1 s, making an ideal uninterrupted test ∼10 min long (579 s of stimulation + 30 s of adaptation = 609 s**/**60 = 10.2 min). In practice, the test lasted ∼20 min (more in older subjects) as many trials were repeated because of fixation breaks, blinks, resting, and some trials lasted 2 s because the subject did not see the target.

The stimulus contrast was calculated as (*L* – *D*)/(*L* + *D*), where *L* is the luminance of the light stimuli (equal for light targets and light background triangles) and *D* is the luminance of the dark stimuli (equal for dark targets and dark background triangles). Because the dark targets have the same luminance as the dark background triangles, the visual responses to the onset of dark targets are strongly dominated by OFF pathways. Similarly, because the light targets have the same luminance as the light background triangles, the visual responses to the onset of light targets are strongly dominated by ON pathways. The absolute luminance difference from midgray was the same for dark and light stimuli. Each subject ran the ON-OFF perimetry test multiple times with eight different contrasts (5%, 6%, 7%, 8%, 9%, 10%, 15%, and 20%), in multiple visits to the laboratory. In the first visit, the subjects ran the ON-OFF perimetry test with the highest stimulus contrast (20% contrast), and, as the subjects became familiar with the test, they ran increasingly lower contrasts. Most subjects were tested with two different contrasts in each visit (∼1 h/visit) and completed the test for the eight different contrasts in four to five visits. One subject required six visits and another, eight visits.

### Data analysis.

All data analyses were performed by selecting stimulus trials with eye movements restricted within ±2.5° radius. Changes in eye position, pupil size, and head position were measured with the eye tracker and the Inertial Measurement Unit sensors of the headset. The eye position was measured in the vertical and horizontal axes separately for each eye. The head position was measured in three different axes, pitch (up to down), yaw (left to right) and roll (tilt left to tilt right while looking at front). The visual field perimetry for each individual subject was measured separately for light and dark stimuli by calculating the percentage of total number of errors, the percentage of missed targets, and average reaction times. The total number of errors included missed targets (i.e., the subject did not see the target) and mislocated targets (i.e., the subject signaled an incorrect target location). For the sake of simplicity, the figures in the article illustrate only the percentages for all errors and missed targets. The percentage of mislocated targets can be inferred by subtracting the percentages of missed targets from total errors and is also reported in the statistical tables. The article reports distribution means for reaction time instead of the medians to facilitate comparisons with previous studies (Komban et al., 2011; Pons et al., 2017, 2019). The distributions of reaction time are moderately skewed (average skewness: for lights, −1.09; for darks, −0.98), and their means are strongly and linearly correlated with the distribution medians (*R*^2^, 0.984; mean RT = 0.91; median RT = 0.01). Therefore, the reader can accurately estimate the median values of the distributions by dividing the reported mean values by 0.91.

Each stimulus polarity was presented three times at each location. Therefore, the percentage of errors could be 0% (zero of three), 33% (one of three), 66% (two of three), and 100% (three of three). The reaction time at each spatial location was calculated by averaging the reaction times of stimulus presentations followed by a correct response. The contrast response functions from each individual subject were calculated by averaging the total errors, missed targets, or reaction times across all spatial positions (excluding the blind spot), separately for each subject. The average contrast response function across all subjects was calculated by averaging the contrast response functions from all individual subjects. The similarity in pupil size and pupil size variations across contrasts were calculated by comparing the means and SDs of pupil size measured within the entire visual field with different contrasts. The eye and head position stability across contrasts were measured by comparing the mean SDs of eye position (horizontal or vertical) and head angles (pitch, yaw, or roll axes) for different contrasts. The time effort devoted to the visual task was also calculated by measuring the time spent by each subject from the first to the last stimulus presentation of the entire visual test, separately for each contrast. The correlations between stimulus contrast and light/dark dominance were measured by calculating light/dark ratios from total errors, missed targets, and reaction time. The light/dark ratio from total errors was calculated as the average percentage of total errors measured with light stimuli divided by the average percentage of total errors measured with dark stimuli. The light/dark ratio for missed targets was calculated similarly but from missed targets instead of total errors. The light/dark ratio for reaction times was calculated as the average reaction time for light stimuli divided by the average reaction time for dark stimuli.

### Experimental design and statistical analysis.

We estimated the number of stimulus trials needed to minimize the average measurement error as follows. First, we calculated the average percentage of total errors across all stimulus positions, separately for each contrast and light–dark polarity in each individual subject (8 contrasts × 2 polarities = 16 contrast–polarity averages). Then, we calculated the same 16 contrast–polarity values from a random sample of trials ranging from a maximum of 500 to a minimum of 100. Then, we subtracted the 16 contrast–polarity averages obtained with all trials from those obtained with a specific subset of trials and averaged the differences across subjects (the same calculation was done for missed targets and reaction times). A similar approach was used to estimate the number of subjects needed to minimize the average measurement error. We first calculated the average percentage of total errors, missed targets, or reaction times across all stimulus positions, contrasts, and subjects. Then, we calculated the difference between the average of all subjects and the average of a random subset of subjects ranging from a maximum of 34 to a minimum of 1. We also calculated the maximum error percentage (E50) and maximum reaction time (T50) measured in 50% of our subjects with each stimulus contrast. All statistical comparisons of medians within this article were performed with two-tailed Wilcoxon tests.

We also used multiple regression analysis to investigate the relative contribution of contrast (eight values from 5% to 20%) and eccentricity (six values from 5° to 30°) to dark/light dominance. For this analysis, we randomly selected cohorts of 21 of 34 subjects and obtained the average total errors and reaction times for each combination of contrast (C) and E (8 × 6 = 48 data values). We then fit the data with three different logarithmic models, one based on eccentricity only (LD = a_e_ + w_e_ log_10_ E), another based on contrast only (LD = a_c_ + w_c_ log_10_ C), and another based on a linear combination of eccentricity and contrast (LD = a_ce_ + w_e_ log_10_ E + w_c_ log_10_ C), where a_e_, a_c_, and a_ce_ are the intercepts for eccentricity, contrast and the eccentricity-contrast combination, respectively, and w_e_ and w_c_ are the weights for eccentricity and contrast, respectively. We repeated the random selection and functional fits 1000 times and measured the distributions of coefficients and goodness of fit (*R*^2^) from the three models. A similar approach was used to model the percentage of errors associated with each stimulus polarity (light or dark). For this analysis, we used the same approach as for light/dark ratios, but we averaged data from three eccentricity ranges (5–10°, 15–20°, and 25–30°) and four contrast ranges (5–6%, 7–8%, 9–10%, and 15–20%) to obtain 12 combinations of eccentricity and contrast. We also fit the equations for log_10_ D (errors with dark stimuli) and log_10_ L (errors with light stimuli) instead of LD. To evaluate statistically significant differences between models (coefficients and *R*^2^), we calculated the probability that the two distributions are different. This probability was calculated as the area of overlap between the distributions divided by their sum (reported as *p* values of Bootstrap tests).

## Results

**Figure 1.**
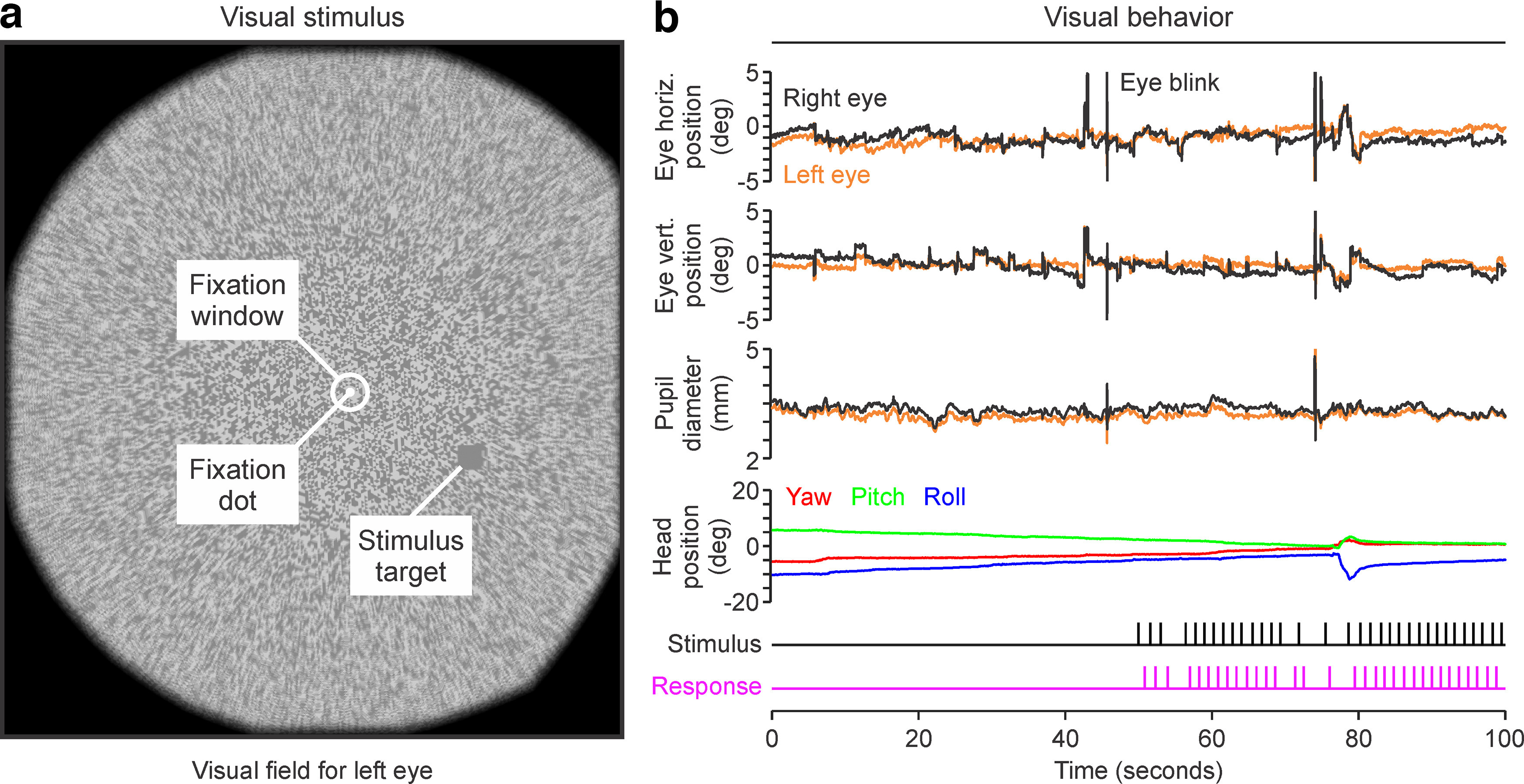
Stimuli and data collection. ***a***, Visual stimuli presented on a head-mounted visual display superimposed on a background made of 50% dark and 50% light triangles. The human subjects fixated a central dot (fixation dot) and pressed a button to indicate whether a square stimulus was presented on the left or right hemifield (stimulus target). Before the button press, eye movements had to be restricted within ±2.5° (fixation window). ***b***, Example recordings from the eye tracker and head motion sensors of the head-mounted visual display. From top to bottom, recordings of horizontal eye position, vertical eye position, and pupil diameter for right eye (black) and left eye (orange), occasionally interrupted by eye blinks. Below the pupil traces, the figure shows recordings from head motion in the yaw (red), pitch (green), and roll (blue) axes, and time stamps for each stimulus presentation (black) and button-press responses (magenta).

**Figure 2.**
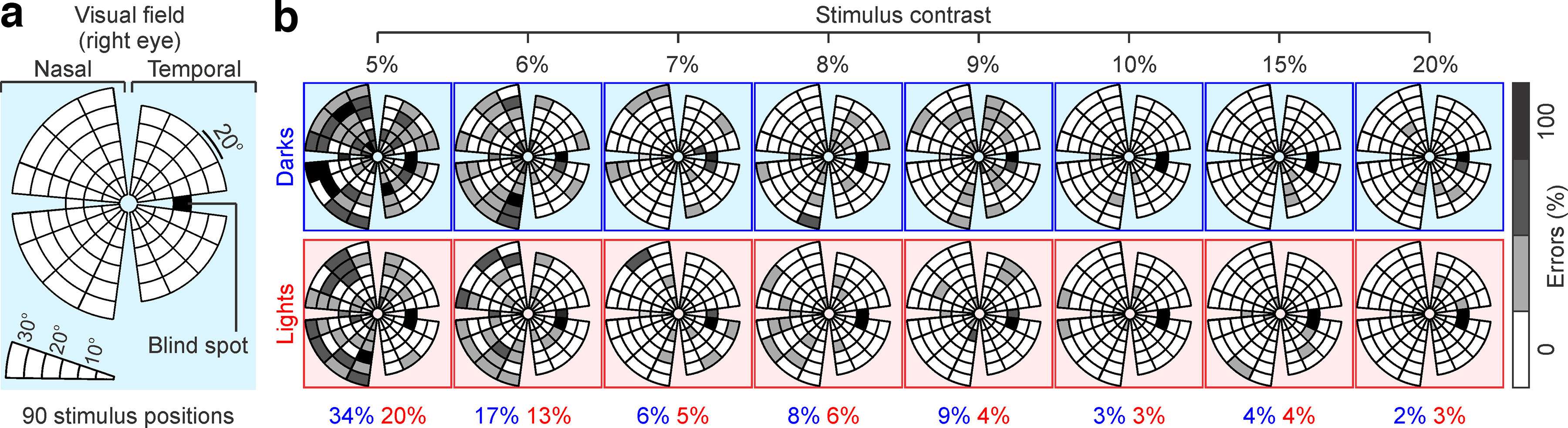
ON-OFF visual field perimetry. ***a***, Visual stimuli were presented in 90 different positions illustrated as circular sectors ranging from 5° to 30° in the radial axis. The visual field was divided into four quadrants by splitting the azimuth axis (nasal or temporal) and elevation axis (upper or lower visual field). Each quadrant was then divided into a number of sectors equal to the number of stimulus positions tested. The sector lines mark the half-distance in angle and radius coordinates between two stimulus positions, except for lines adjacent to a quadrant limit, which mark half the distance between the limit and the target position. ***b***, Analysis of ON-OFF perimetry in an example subject. The visual field was mapped with dark targets (top, blue frames) or light targets (bottom, red frames) of 8 different contrasts (labels at the top). The grayscale illustrates the percentage of detection errors (white, 0%; black, 100%). The average error percentage for each stimulus contrast is shown at the bottom of the figure separately for dark (blue) and light (red) stimuli.

We measured the ability of human subjects to search for light and dark stimuli within the central 30° of the visual field while fixating on a dot at the center of a head-mounted visual display (Fig. 1*a*). The subjects signaled the location of the target (left or right) by pushing a button while fixating within a ±2.5° window. Visual behavior was continuously monitored (Fig. 1*b*; see Materials and Methods) and visual performance analyzed separately for light and dark stimuli to estimate the function of ON and OFF visual pathways across the visual field (Fig. 2*a*).

### ON-OFF visual field perimetry

**Figure 3.**
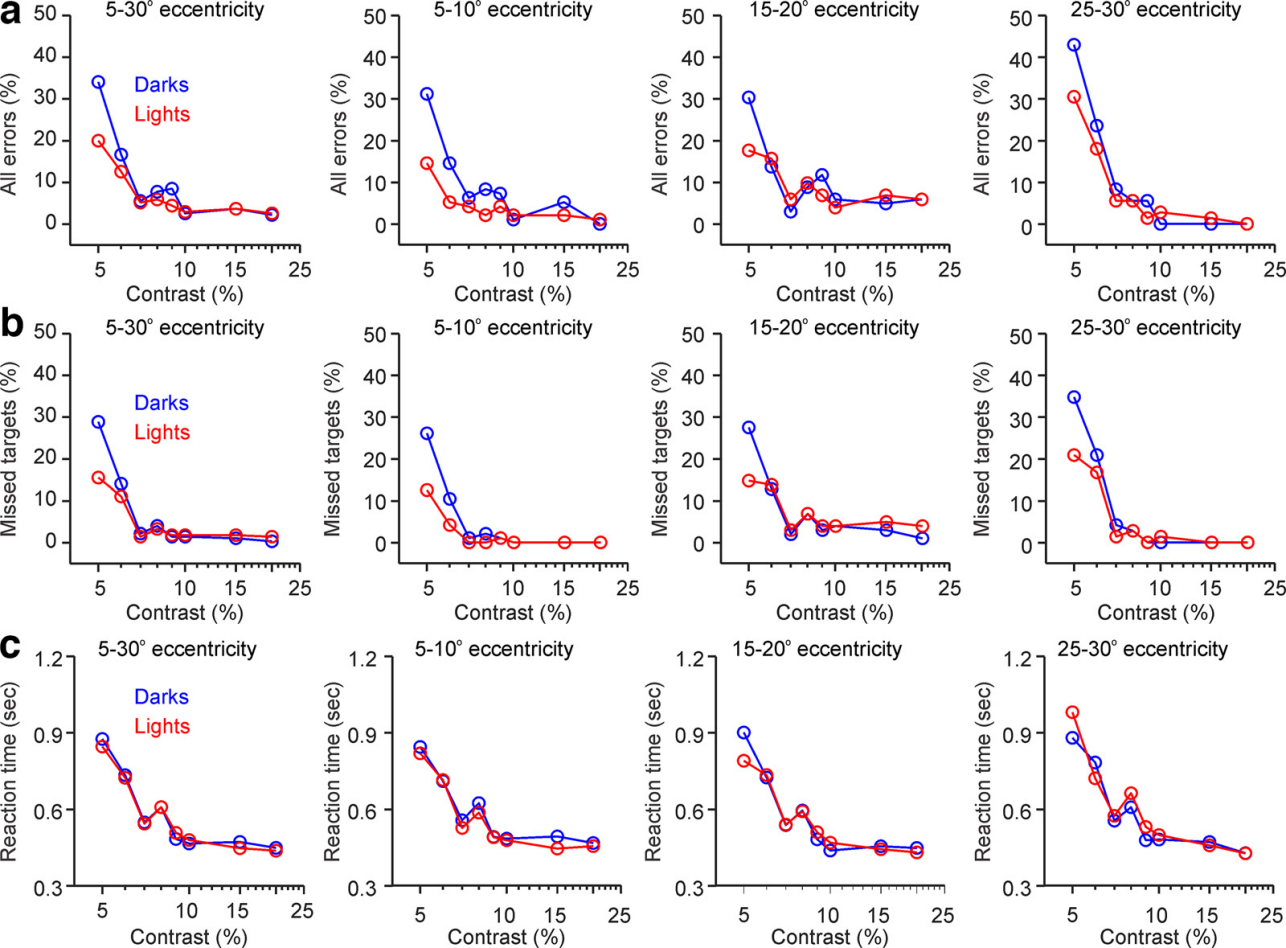
ON-OFF contrast–response functions of an example human subject (same subject as in Fig. 2*b*). ***a***, Percentage of all errors, including missed targets (i.e., the target is present, but the subject signals that there is no target) and mislocated targets (e.g., the target appears on the left hemifield but the subject signals a target in right hemifield). From left to right, the figure shows the ON-OFF contrast response function averaged across all tested eccentricities (5–30°) and at specific eccentricity ranges (5–10°, 15–20°, and 25–30°), measured separately for dark (blue) and light targets (red). ***b***, Same as ***a*** for missed targets. ***c***, Same as ***a*** for reaction time.

The subjects performed this ON-OFF perimetry test multiple times, each time with a different stimulus contrast (Fig. 2*b*). As expected, increasing the contrast reduced the percentage of all errors, including missed targets (e.g., the subject did not see the target) and mislocated targets (e.g., the subject pressed the left button when a target was on the right hemifield or vice versa). For example, in the subject illustrated in Figure 2*b*, increasing the contrast from 5% to 20% reduced the percentage of errors by one order of magnitude and the reaction time by nearly two times across all eccentricities (Fig. 3*a–c*).

Whereas the most obvious effect of increasing stimulus contrast was a reduction in errors and reaction times, the reduction was not the same for light and dark stimuli. When the contrast was low (5%), the subject illustrated in Figure 2 made more errors when searching for dark stimuli than for light stimuli (Fig. 3*a*,*b*), and the same was true for 27 of 34 subjects (27 vs 7; *p* = 0.0006, χ^2^ test). Conversely, when the contrast was high (20%), the subject illustrated in Figure 2 made slightly more errors when searching for light than dark stimuli (Fig. 3*a*,*b*) and the same was true for 25 of 34 subjects (25 vs 9; *p* = 0.0061, χ^2^ test). At 5% contrast, most subjects responded faster to light than dark stimuli (29 vs 5; *p* < 0.00,001, χ^2^ test), whereas, at 20% contrast, most subjects responded faster to dark stimuli than light stimuli, although the difference in subject number was not significant at high contrasts (20 vs 14; *p* = 0.3035, χ^2^ test). Significant dark–light differences were difficult to demonstrate in each individual subject because statistical comparisons of error percentages require a larger number of stimulus trials. However, at 5% contrast, the reaction time was significantly faster for light stimuli than dark stimuli in 12 individual subjects (*p* < 0.05 for each individual subject; *p* value range, 0.000001–0.02, Wilcoxon tests).

**Figure 4.**
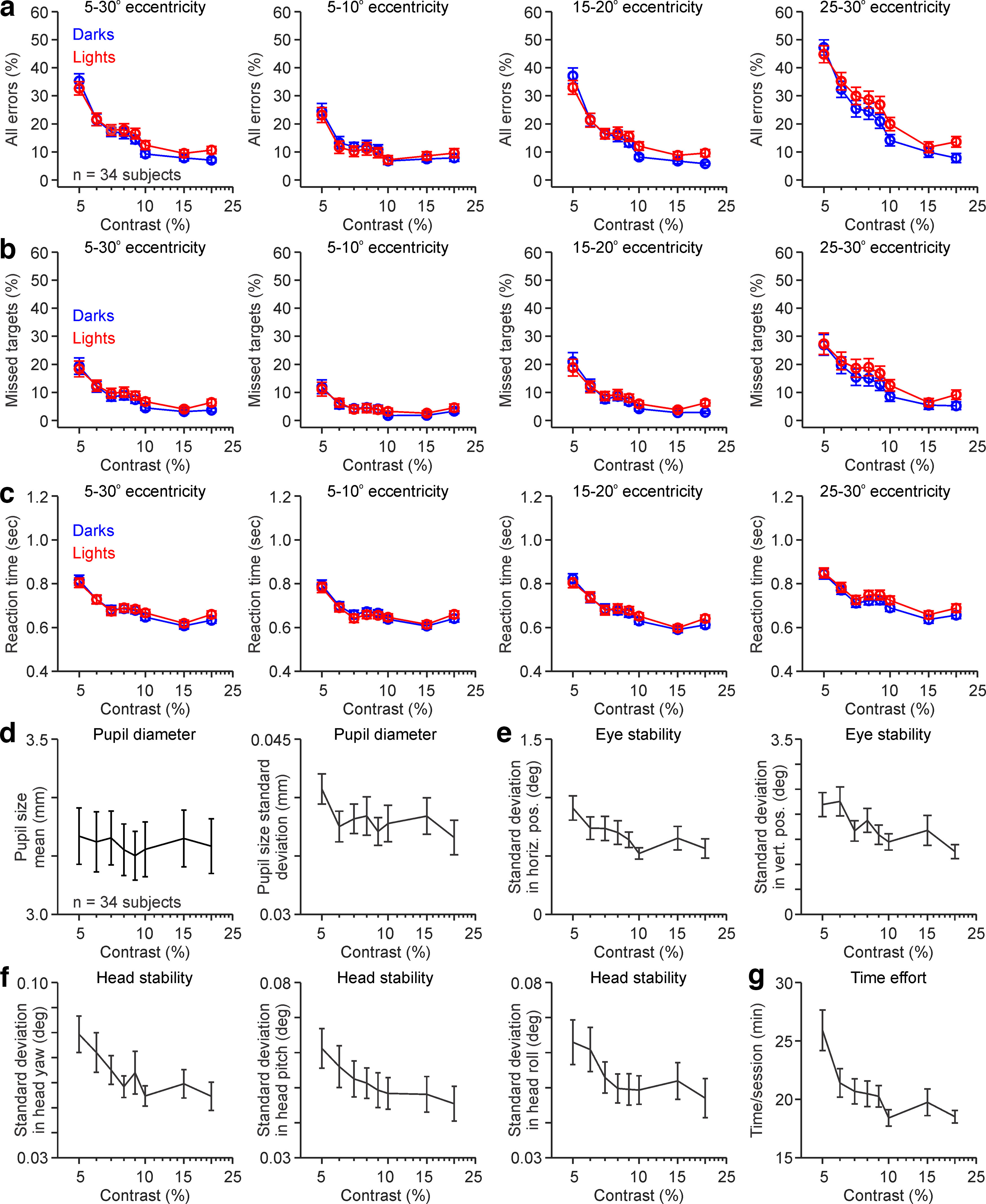
ON-OFF contrast-response functions of subject average. ***a–c***, Contrast-response functions for darks (blue) and lights (red) calculated from the percentage of all errors (***a***), missed targets (***b***), and reaction times (***c***) at different eccentricity ranges. ***d***, Average (left) and SDs (right) of pupil diameters measured in 34 subjects. In each subject, each pupil mean and SD was measured during the entire stimulus presentation, separately for each contrast. ***e***, Same as ***d*** for horizontal (left) and vertical (right) eye position. ***f***, Same as ***d*** for head yaw (left), pitch (middle), and roll (right). ***g***, Time spent in each task session for each stimulus contrast. Error bars are standard error of means (SEM).

As in the example subject, increasing the stimulus contrast reduced the percentage of errors and reaction time in the subject average (Fig. 4; *n* = 34 subjects). Increasing the contrast from 5% to 20% reduced the average total errors by 3.6 times (Fig. 4*a*; 33.6 ± 14.5% vs 9.3 ± 7.1%; *p* < 0.00,001, Wilcoxon test), the average missed targets by 3.4 times (Fig. 4*b*; 18.4 ± 17.0% vs 5.5 ± 5.9%; *p* < 0.00001, Wilcoxon test), and the average reaction time by 1.3 times (Fig. 4*c*; 0.81 ± 0.13 s vs 0.65 ± 0.09 s; *p* < 0.00,001, Wilcoxon test). Increasing the contrast from 5% to 20% also improved the visuomotor stability (Fig. 4*d–f*) and reduced the time needed to complete the test (Fig. 4*g*). At the lowest contrast of 5%, the pupil, eye, and head were reasonably stable, and the changes limited to <45 μm for pupil size (Fig. 4*d*), <2.5° for eye position (Fig. 4*e*), and <0.1° for head position (Fig. 4*f*). However, at 20% contrast, the stability increased further (i.e., variability decreased) by 1.1 times for pupil size (40.8 ± 10.2 μm vs 36.6 ± 11.8 μm; *p* = 0.039, Wilcoxon test), 1.7 times for eye position (1.56 ± 1.60° vs 0.91 ± 0.97°; *p* < 0.0001, Wilcoxon test), and 1.4 times for head position (0.07 ± 0.04° vs 0.05 ± 0.03°; *p* < 0.0001, Wilcoxon test). Increasing the contrast also reduced the time to complete the test by 1.4 times (25.93 ± 9.72 min vs 18.53 ± 2.99 min; *p* = 0.0005, Wilcoxon test).

As with the example subject, stimulus contrast also affected the visibility of light and dark stimuli differently in the subject average. At the lowest contrast (5%), the percentage of errors was lower (Fig. 4*a*,*b*) and the reaction time faster (Fig. 4*c*) for light stimuli than for dark stimuli. Conversely, at the highest contrast (10–20%), the percentage of errors was higher (Fig. 4*a*,*b*) and the reaction time was slower (Fig. 4*c*) for light stimuli than for dark stimuli (Table 1, see statistical comparisons).

**Table 1.** Comparison of visual detection for light and dark stimuli at different contrast ranges

Eccentricity (°)	Contrast range (%)	Contrast polarity	All errors (%)	Mislocation errors (%)	Missed targets (%)	Reaction time (ms)
5–30	5–20	Darks	16.08 ± 12.89	7.68 ± 7.25	8.40 ± 10.49	682.5 ± 119.6
		Lights	17.37 ± 12.90	7.80 ± 6.84	9.58 ± 10.93	689.7 ± 119.2
		*p* value	***p* < 0.00001**	*p* = 0.1652	***p* < 0.00001**	***p* = 0.00042**
	5	Darks	34.86 ± 14.82	15.96 ± 10.99	18.90 ± 17.44	815.4 ± 136.4
		Lights	32.42 ± 14.25	14.50 ± 10.16	17.93 ± 16.89	804.2 ± 130.6
		*p* value	***p* = 0.0035**	***p* = 0.0077**	*p* = 0.3263	***p* = 0.0426**
	10–20	Darks	8.45 ± 6.31	4.38 ± 3.44	4.07 ± 4.58	627.8 ± 87.3
		Lights	11.22 ± 8.61	5.13 ± 4.06	6.86 ± 6.67	647.3 ± 96.6
		*p* value	***p* < 0.00001**	***p* = 0.00039**	***p* < 0.00001**	***p* < 0.00001**

From left to right, eccentricity range (all measurements averaged across all eccentricities), contrast range (measurements averaged across 5–20%, 5%, or 10–20% contrast), contrast polarity (dark or light), errors (all, mislocation, missed), and reaction times. The numbers within each cell are mean ± SD and *p* values that show there is no difference between darks and lights. Significant *p* values (<0.05) are highlighted in bold.

### The visual dominance of the OFF pathway increases with luminance contrast

**Figure 5.**
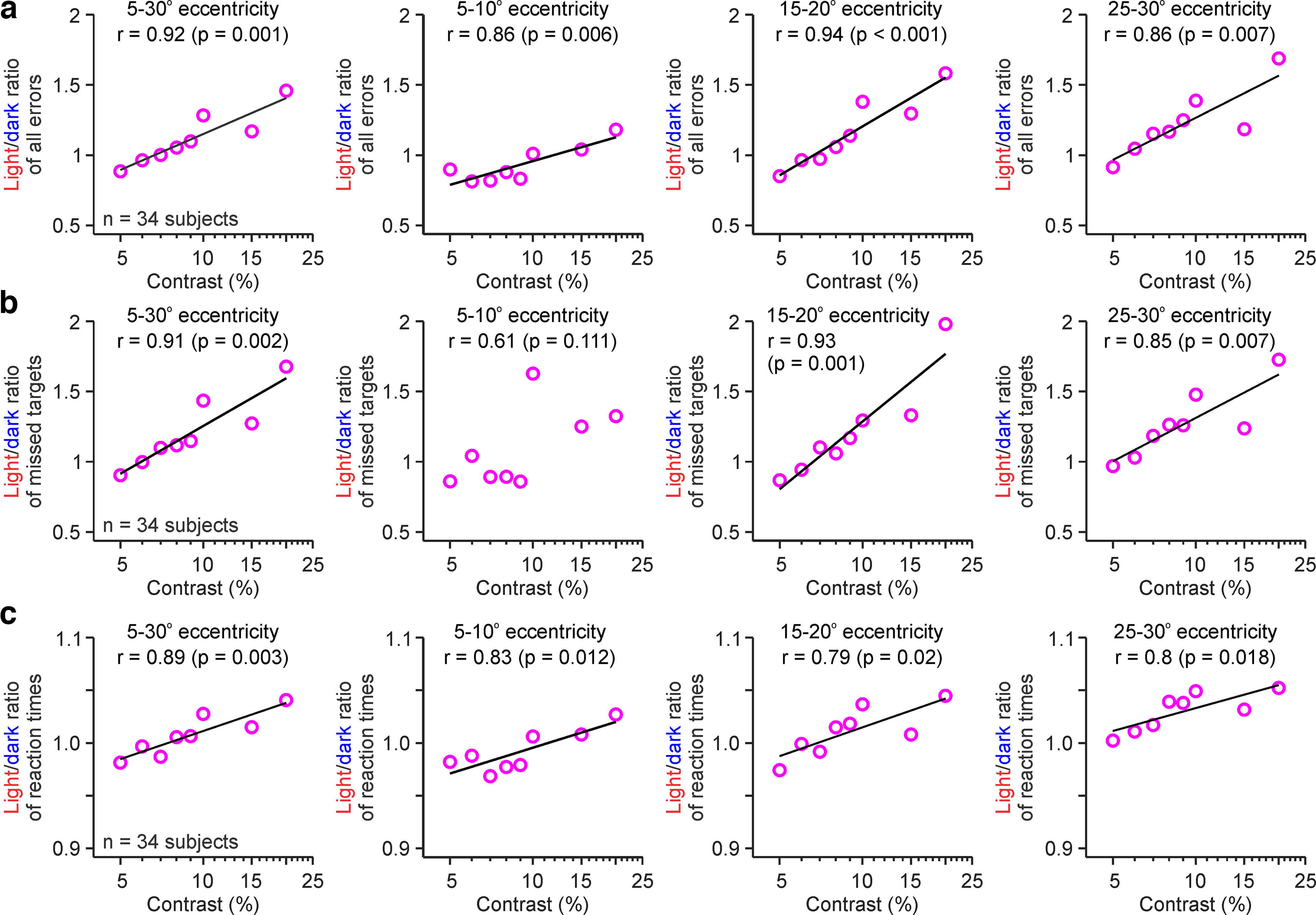
Dominance of dark stimuli increases with contrast in human vision. ***a***, The ratio of errors at detecting light versus dark stimuli (light/dark ratio of all errors) was strongly correlated with stimulus contrast at all visual eccentricities tested. The labels at the top report the eccentricity range (from left to right: 5–30°, 5–10°, 15–20°, and 20–30°), goodness of fit (*R*^2^) and probability that the correlation is because of chance. ***b***, Same as ***a*** for light/dark ratio of missed targets. Regression lines only showed for significant correlations. ***c***, Same as a for light/dark ratio of reaction time.

The dependence of the light/dark dominance on contrast was very robust and could be quantified by calculating light/dark ratios of total errors (Fig. 5*a*), missed targets (Fig. 5*b*), and reaction time (Fig. 5*c*). Nearly all light/dark ratios at all eccentricity ranges were strongly and significantly correlated with stimulus contrast. The correlations between contrast and the light/dark ratios could be accurately fit with logarithmic functions described as LD = intercept + slope × log_10_ C, where “intercept” is the LD value at 1% contrast and “slope” is the rate of ratio change with contrast. Within the 5–30° eccentricity range, the logarithmic equations were LD = 0.4 + 0.8 log_10_ C for total errors (*R*^2^ = 0.918, *p* = 0.001), LD = 0.2 + 1.1 log_10_ C for missed targets (*R*^2^ = 0.908, *p* = 0.002), and LD = 0.9 + 0.1 log_10_ C for reaction times (*R*^2^ = 0.891, *p* = 0.003).

### The visual dominance of the OFF pathway changes with visual eccentricity

**Figure 6.**
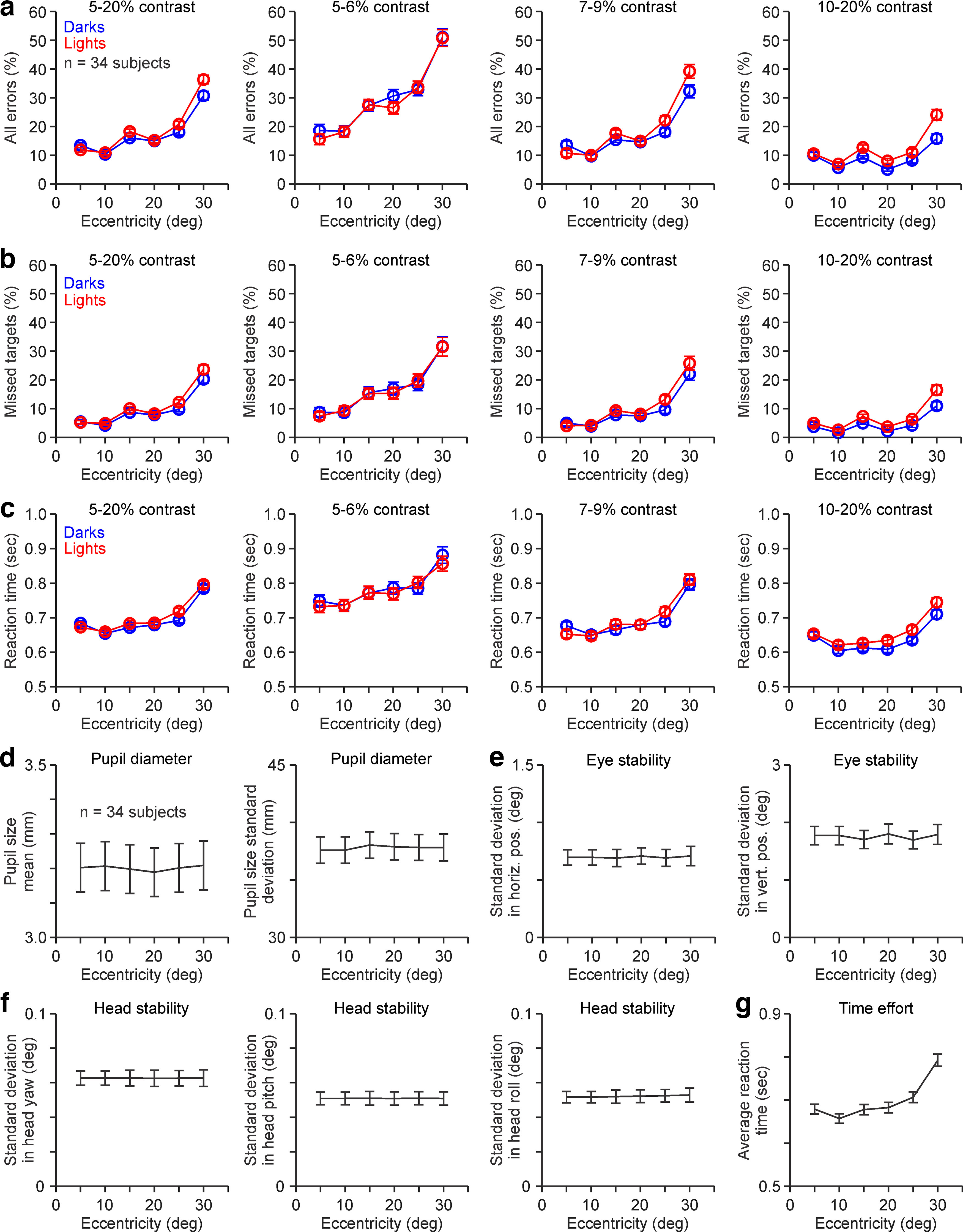
ON-OFF eccentricity–response functions of subject average. ***a–c***, Eccentricity–response functions for darks (blue) and lights (red) calculated from the percentage of all errors (***a***), missed targets (***b***), and reaction times (***c***). ***d***, Average (left) and SDs (right) of pupil diameters measured in 34 subjects. In each subject, each pupil mean and SD was measured during the entire stimulus presentation, separately for each eccentricity range. ***e***, Same as ***d*** for horizontal (left) and vertical (right) eye position. ***f***, Same as ***d*** for head yaw (left), pitch (middle), and roll (right). ***g***, Response time spent for each stimulus eccentricity averaged across all stimulus targets and subjects. Error bars are SEM.

The visibility of light and dark targets also changed with visual eccentricity. As the stimuli became farther from the fovea, the total number of errors (Fig. 6*a*), missed targets (Fig. 6*b*), and reaction times (Fig. 6*c*) increased, and the increase was most pronounced when the stimulus contrast was low. At the lowest contrasts (5–6%), increasing eccentricity from 5° to 30° raised the total percentage of errors by three times (17.2 ± 16.4% vs 51.0 ± 21.6%; *p* < 0.00001, Wilcoxon test), the missed targets by 3.9 times (8.1 ± 12.8% vs 31.7 ± 27.1%; *p* < 0.00001, Wilcoxon test), and the reaction time by 1.2 times (0.74 ± 0.14 s vs 0.87 ± 0.19 s for reaction time; *p* < 0.00001, Wilcoxon test), an increase that would have been even more pronounced if the stimulus size was the same at all eccentricities (the stimulus size was 1.6 times larger at 30° than at 5°; see Materials and Methods). Similarly, at the highest contrast (10–20%), increasing eccentricity from 5° to 30° raised the total errors by 1.9 times (10.3 ± 11.5% vs 20 ± 18.2%; *p* < 0.00001, Wilcoxon test), the missed targets by 3.2 times (4.4 ± 8.5% vs 13.8 ± 16.1%; *p* < 0.00001, Wilcoxon test), and the reaction time by 1.1 times (0.65 ± 0.1 s vs 0.73 ± 0.13 s; *p* < 0.00001, Wilcoxon test). At the highest contrasts (10–20%), increasing eccentricity from 5° to 10° also reduced the percentage of errors (10.3 ± 11.5% vs 6.4 ± 6.8%; *p* < 0.00001, Wilcoxon test) and reaction times (0.65 ± 0.1 s vs 0.61 ± 0.1 s; *p* < 0.00,001, Wilcoxon test), probably because the size was larger at 10° than at 5° of eccentricity (3° vs 2.5°). Across visual eccentricities and contrasts, the average pupil size (Fig. 6*d*), eye position (Fig. 6*e*), and head position (Fig. 6*f*) were very similar. Only the average reaction time increased when the stimuli became farther away from the fovea (Fig. 6*g*).

**Figure 7.**
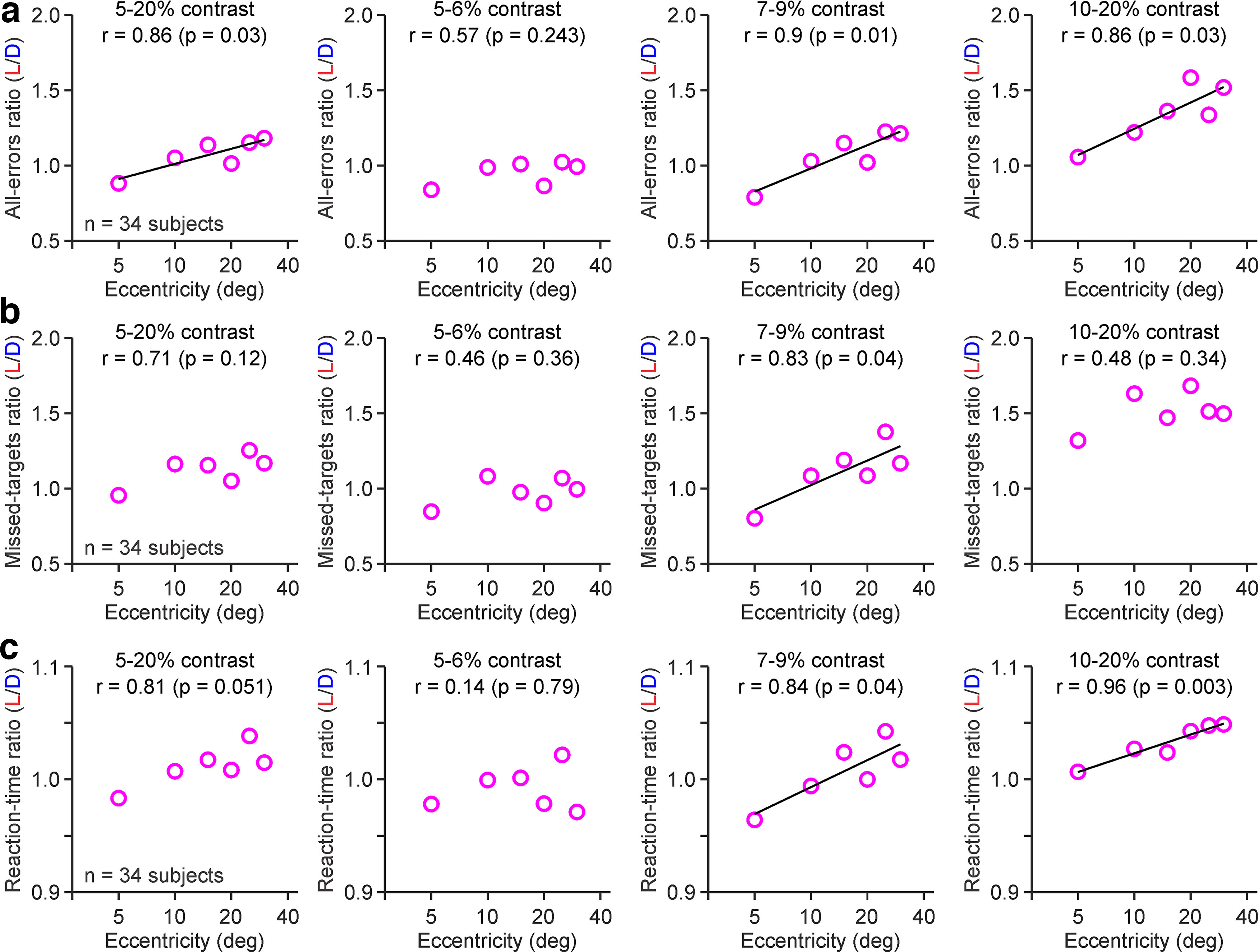
Dominance of dark stimuli increases with eccentricity in human central vision. ***a***, The ratio of errors at detecting light versus dark stimuli (light/dark ratio of all errors) was significantly correlated with the logarithm of eccentricity at contrasts >6%. The labels at the top report the contrast range (from left to right: 5–20%, 5–6%, 7–9%, and 10–20%), goodness of fit (*R*^2^) and probability that the correlation is because of chance. ***b***, Same as ***a*** for light/dark ratio of missed targets. ***c***, Same as ***a*** for light/dark ratio of reaction time.

As with contrast, visual eccentricity affected differently the visibility of light and dark targets. At the lowest contrasts (5–6%), the errors and reaction time were approximately similar for light than for dark targets at all eccentricities (Fig. 6*a–c*; 5–6% contrast panel). However, as the contrast became higher, the total number of errors, missed targets, and reaction times all increased with eccentricity for light stimuli more than dark stimuli, making the light/dark ratios larger at high eccentricities (Fig. 7*a–c*, Table 2, statistical comparisons). At 7–9% contrasts, visual eccentricity was significantly correlated with the three light/dark ratios calculated from the total number of errors (Fig. 7*a*), missed targets (Fig. 7*b*), and reaction times (Fig. 7*c*). The light/dark ratios averaged across all contrasts increased significantly less from 20° to 30° than from 5° to 15° eccentricity for both total errors (0.13 ± 1.26 vs 0.45 ± 1.35; *p* = 0.019, Wilcoxon test) and missed targets (0.15 ± 1.34 vs 0.45 ± 1.74; *p* = 0.016, Wilcoxon test). Therefore, light/dark ratios in human vision may not increase further (or even decrease) at large eccentricities, as demonstrated in carnivores and rodents (Jin et al., 2008; Williams et al., 2021).

**Table 2.** Comparison of visual detection for light and dark stimuli with low or high contrast at different eccentricity ranges

Contrast range (%)	Eccentricity (°)	Contrast polarity	All errors (%)	Mislocation errors (%)	Missed targets (%)	Reaction time (ms)
5–6	5–30	Darks	29.86 ± 21.26	13.16 ± 12.01	16.70 ± 19.71	785.2 ± 160.7
		Lights	28.76 ± 21.12	12.30 ± 11.20	16.46 ± 19.38	778.1 ± 156.5
		*p* value	***p* = 0.0337**	***p* = 0.0426**	*p* = 0.7108	*p* = 0.2250
	5–10	Darks	18.53 ± 16.39	9.84 ± 9.60	8.69 ± 12.69	742.7 ± 140.4
		Lights	16.93 ± 15.67	8.55 ± 8.97	8.38 ± 12.39	734.4 ± 141.0
		*p* value	***p* = 0.0493**	***p* = 0.0171**	*p* = 0.8005	*p* = 0.0765
	25–30	Darks	42.06 ± 22.53	16.96 ± 13.30	25.09 ± 24.16	833.7 ± 177.4
		Lights	42.29 ± 22.52	16.60 ± 12.73	25.68 ± 23.76	829.3 ± 165.0
		*p* value	*p* = 0.8045	*p* = 0.9608	*p* = 0.3378	*p* = 0.7115
10–20	5–30	Darks	9.05 ± 10.33	4.41 ± 4.95	4.63 ± 8.33	636.9 ± 105.8
		Lights	12.26 ± 13.24	5.33 ± 5.82	6.93 ± 10.83	658.0 ± 116.3
		*p* value	***p* < 0.00001**	***p* = 0.000080**	***p* < 0.00001**	***p* < 0.00001**
	5–10	Darks	7.86 ± 8.50	5.18 ± 4.79	2.67 ± 5.52	627.2 ± 96.4
		Lights	8.78 ± 10.65	4.98 ± 4.85	3.80 ± 7.82	637.6 ± 101.8
		*p* value	*p* = 0.0523	*p* = 0.3228	***p* = 0.000425**	***p* = 0.0060**
	25–30	Darks	12.06 ± 14.05	4.44 ± 6.04	7.62 ± 11.97	673.1 ± 118.5
		Lights	17.59 ± 16.89	6.13 ± 7.22	11.45 ± 14.62	705.6 ± 132.2
		*p* value	***p* < 0.00001**	***p* = 0.00029**	***p* < 0.00001**	***p* < 0.00001**

From left to right, contrast range (measurements averaged across 5–6% contrasts; measurements averaged across 10–20% contrast), eccentricity range (measurements averaged across 5–10°, 15–20°, or 25–30°). Same format as in Table 1.

As for the correlations of light/dark ratios with contrast, the correlations with eccentricity could be accurately fit with logarithmic functions described as LD = intercept + slope × log_10_ E, where intercept is the LD value at 1° eccentricity and slope is the rate of ratio change with eccentricity. Within the 7–9% contrast range, the logarithmic equations were LD = 0.5 + 0.5 log_10_ E for total errors (*R*^2^ = 0.903, *p* = 0.01), LD = 0.5 + 0.5 log_10_ E for missed targets (*R*^2^ = 0.832, *p* = 0.04), and LD = 0.9 + 0.1 log_10_ E for reaction time (*R*^2^ = 0.839, *p* = 0.04).

### Reliability of ON-OFF perimetry measurements

**Figure 8.**
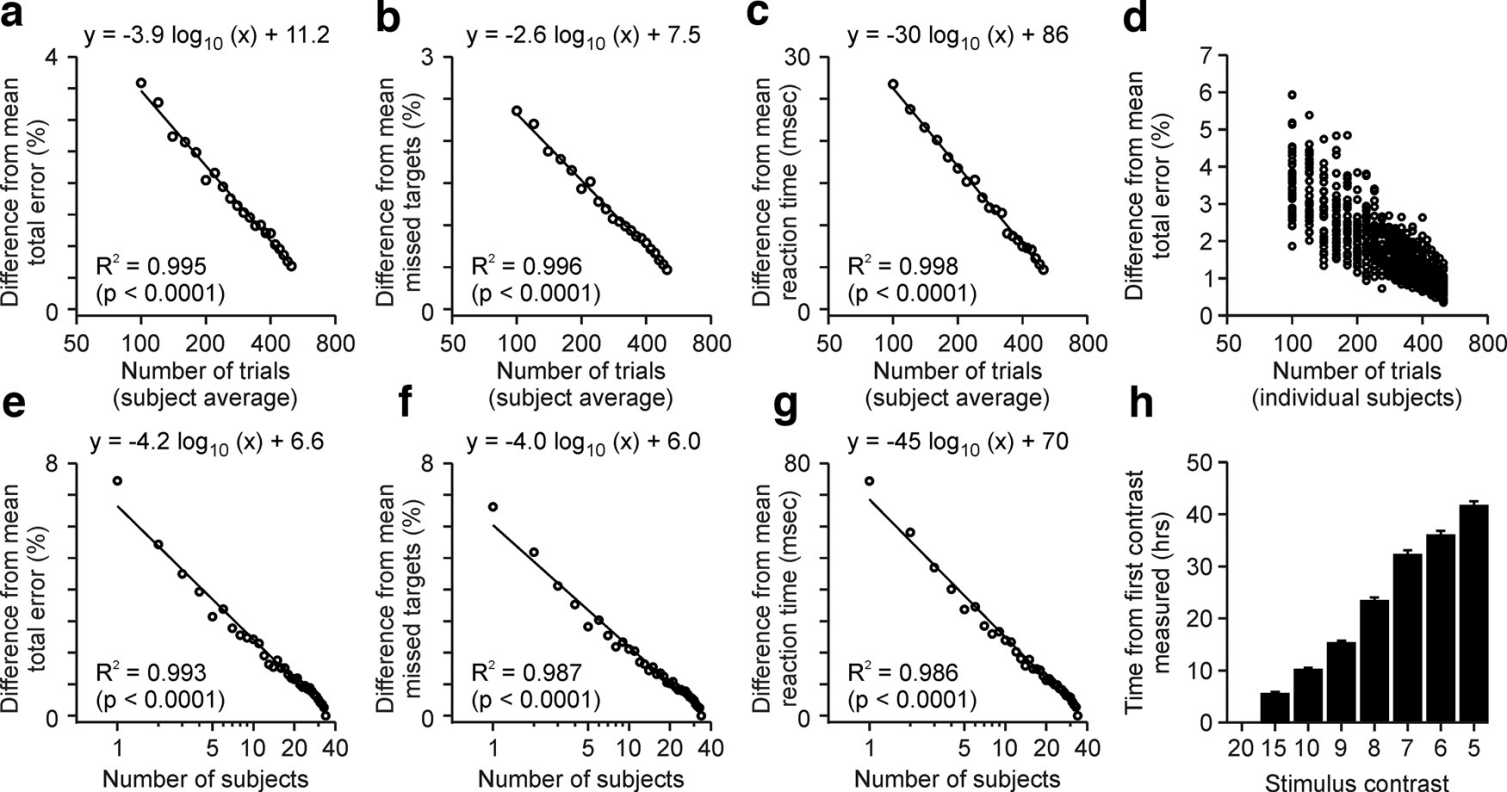
Reliability of ON-OFF perimetry measurements. ***a–c***, The means of total error (***a***), missed targets (***b***), and reaction times (***c***) decrease logarithmically with the number of stimulus trials. Decreasing the number of trials from 579 to 100 only causes a modest reduction in visual performance (<4% of total error, <3% of missed targets, <30 ms of reaction times). Notice that the maximum *x* value plotted is 500 trials. The *y* value is zero at *x*: 579 trials. ***d***, Same as *a* for individual subjects. Each circle illustrates the average total error from each subject measured with the number of trials shown at the *x*-axis. ***e–g***, The means of total error (***e***), missed targets (***f***), and reaction times (***g***) decrease logarithmically with the number of subjects. Decreasing the number of subjects from 30 to 1 causes a relatively modest reduction in average visual performance (<8% of total error and missed targets; <80 ms of reaction time). ***h***, Average time between contrast measurements, from the highest contrast that was tested first (20%) to the lowest contrast that was tested last (5%). Error bars are SEM.

The measurements of ON-OFF perimetry were highly repeatable across stimulus trials and subjects. As expected, testing the visual field with more stimulus trials reduced the average of total errors (Fig. 8*a*), missed targets (Fig. 8*b*), and reaction times (Fig. 8*c*). As the number of stimulus trials increased, subset averages calculated with a subset of stimulus trials became closer to the overall average calculated with all stimulus trials (for more details, see Materials and Methods). Consequently, the difference between subset and overall averages became closer to zero (Fig. 8*a–c*).

Increasing the number of trials also made the test longer, reducing its potential for clinical applications. Therefore, it is important to quantify the relation between the number of stimulus trials and test reliability. Our analysis indicates that ON-OFF perimetry can be reasonably accurate with just 100 stimulus trials. In fact, doubling the number of trials from 100 to 200 decreased the mean error by just 1.2% (Fig. 8*a*), missed targets by 0.8% (Fig. 8*b*), mean reaction time by 9 ms (Fig. 8*c*), and the error range across individual subjects by 1.4% (Fig. 8*d*; 1.9–5.9% vs 1.2–3.8%). Increasing the number of subjects from 1 to 10 also reduced the mean error by just 4.2%, missed targets by 4%, and reaction times by 45 ms. Therefore, these results indicate that ON-OFF perimetry could potentially be used to evaluate the visual function of single subjects with a limited number of stimulus trials. The high reliability of ON-OFF perimetry in part may be because of the order of contrasts tested, which allowed subjects to practice with high-contrast targets before they were exposed to targets of increasingly lower contrast (Fig. 8*h*). Therefore, the order of contrasts may be an important parameter to consider in the design of future clinical applications.

**Figure 9.**
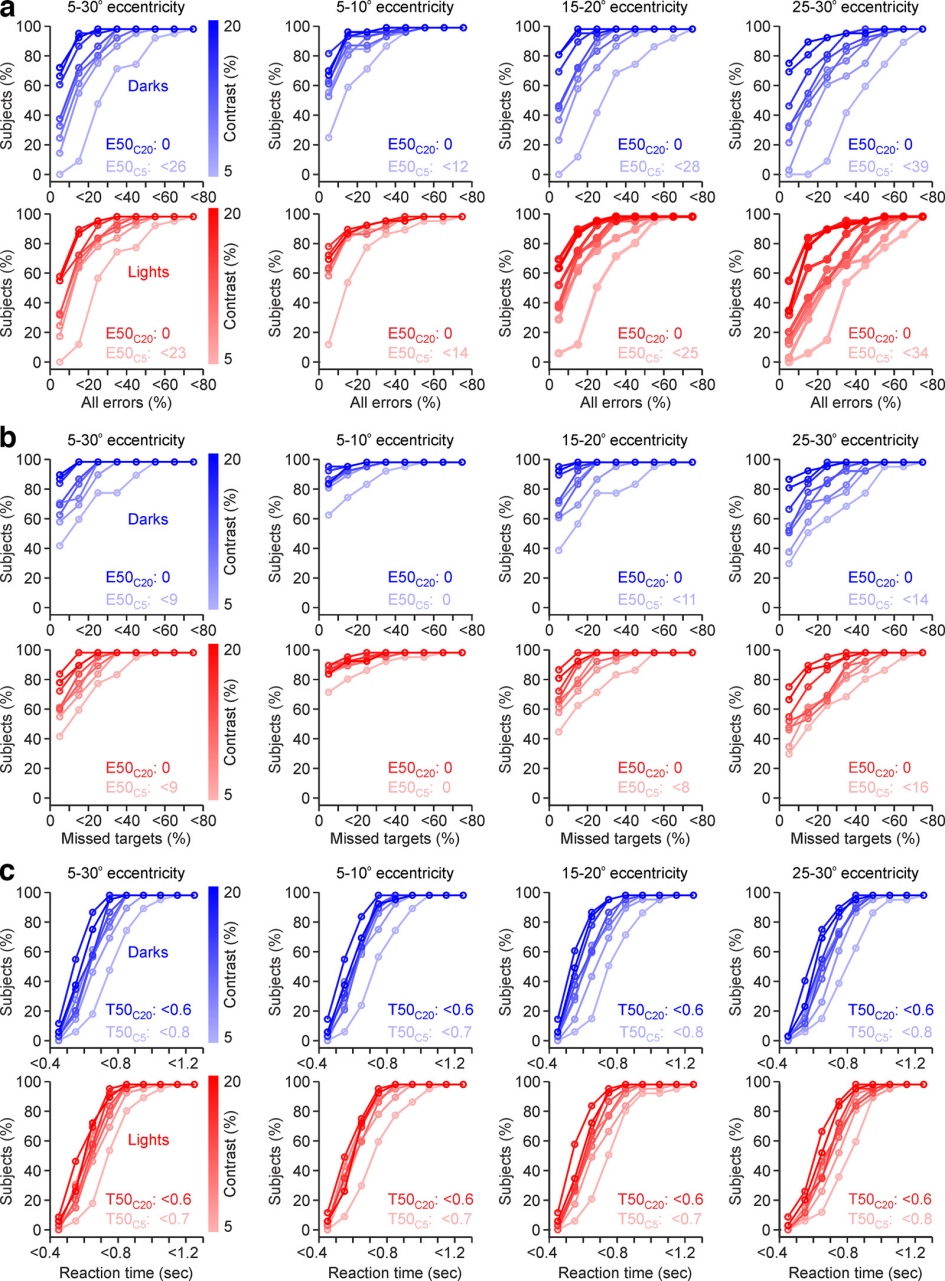
Functions of visual performance from accumulated subject percentage. ***a***, Functions of all errors for dark (top, blue) and light stimuli (bottom, red), measured at different eccentricity ranges (from left to right: 5–30°, 5–10°, 15–20°, and 25–30°). High contrasts are illustrated by saturated colors (blues and reds with high color contrast) and low contrasts illustrated by nonsaturated colors (blues and reds with low color contrast). The E50_C20_ value is 0. At 5% contrast, the accumulated error of 50% of the subjects (E50_C5_) increases with eccentricity. ***b***, Same as ***a*** for missed targets. ***c***, Same as ***a*** for reaction time.

A more detailed quantification of test reliability can be obtained by measuring functions of accumulated error and reaction time across all subjects. These analyses demonstrate that most subjects make a very limited number of errors when searching for high-contrast stimuli. At C20, 50% of subjects made no errors at any eccentricity (Fig. 9*a*; E50_C20_, 0) and, only when the contrast was reduced to 5%, the number of errors increased, and mostly at high eccentricities. For example, at 5% contrast, 50% of the subjects made 12–14% errors at 5−10° (Fig. 9*a*; E50_C5_, <12–14%) and almost three times more at 25−30° eccentricity (Fig. 9*a*; E50_C5_, <34–39%). Similar results could be demonstrated for missed targets (Fig. 9*b*) and reaction times (Fig. 9*c*). For example, at 20% contrast, 50% of subjects missed no targets (Fig. 9*b*; E50_C20_, 0) and were able to locate a target within <600 ms at any eccentricity (Fig. 9*c*; E50_C20_, <0.6 s). However, as the contrast decreased to 5%, they needed 700 ms to search for targets at 5−10° eccentricity (Fig. 9*c*; E50_C5_, <0.7 s). The number of errors, missed targets, and reaction times also increased with age at all contrasts. However, although highly significant, the correlations between age and visual performance were relatively weak because the individual variability in visual performance was higher than the variation with age (*r* = 0.25 for total errors, *r* = 0.35 for missed targets, *r* = 0.43 for reaction time, *p* < 0.00,001 for all).

### Relative contribution of contrast and eccentricity to light/dark dominance

**Figure 10.**
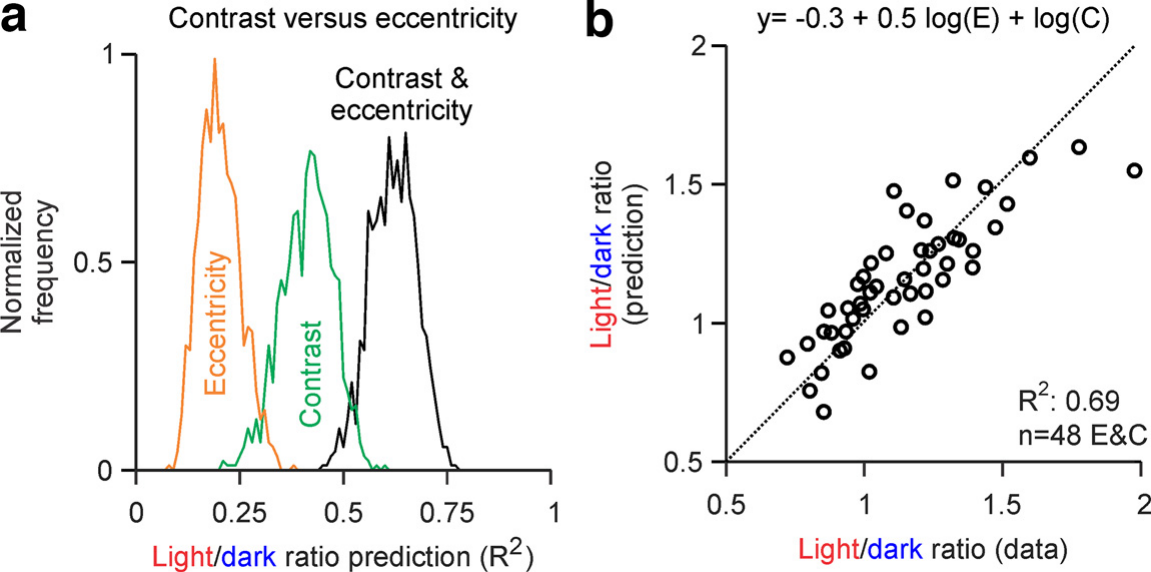
Relative contribution of contrast and eccentricity to light/dark dominance. ***a***, Goodness of fit (*R*^2^) of three models explaining the variance in light/dark dominance with eccentricity only (orange), contrast only (green), or a linear combination of eccentricity and contrast (black). ***b***, A model that weights C two times more than E fits reasonably well the data averaged across all subjects.

Our results demonstrate that the light/dark ratio of visual dominance changes with both contrast and eccentricity. To quantify the relative contribution of these two stimulus parameters, we performed multiple regression analysis. We randomly selected 21 of 34 subjects and calculated the average error for each combination of eccentricity and contrast (48 data values). We then fit the data with a logarithmic model described as LD = a + w log_10_ b, where b is the stimulus variable (eccentricity or contrast). We tested three different models, using eccentricity only, contrast only, or a linear combination of both eccentricity and contrast (Fig. 10*a*; for details, see Materials and Methods). The results from multiple regression analysis demonstrate that eccentricity explains 21% of the variance in light/dark dominance (*R*^2^, 0.21 ± 0.05), contrast explains 42% (*R*^2^, 0.42 ± 0.06), and the linear combination of contrast and eccentricity explains 63% (*R*^2^, 0.63 ± 0.05). Therefore, the linear combination of contrast and eccentricity explains three times more variance than eccentricity alone (*R*^2^, 0.63 vs 0.21; *p* = 0.001, Bootstrap test) and 1.5 times more variance than contrast alone (*R*^2^, 0.63 vs 0.42; *p* = 0.0395, Bootstrap test). Moreover, contrast alone explains two times more variance than eccentricity alone (*R*^2^, 0.42 vs 0.21; *p* = 0.049, Bootstrap test). The model weights were also two times larger for contrast than for eccentricity (0.96 ± 0.14 vs 0.48 ± 0.07; *p* = 0.01, Bootstrap test), and this weighted combination of contrast and eccentricity provided an accurate fit of the data (Fig. 10*b*). Therefore, we conclude that contrast affects light/dark dominance more than eccentricity.

We used a similar approach to estimate the relative contribution of eccentricity and contrast in the detection of dark or light stimuli. For this analysis, we averaged data from three eccentricity ranges (5–10°, 15–20°, and 25–30°) and four contrast ranges (5–6%, 7–8%, 9–10%, and 15–20%) to obtain 12 data combinations of eccentricity and contrast. We then randomly selected 21 of 34 subjects and fit the data 1000 times with similar functions to those used for light/dark ratios. The logarithmic functions revealed by this analysis explain 87% of the error variance for Ds and 89% for Ls. The functions for dark stimuli (log_10_ D = 1.6 + 0.5 log_10_ E – 1.2 log_10_ C) and light stimuli (log_10_ L = 1.1 + 0.8 log_10_ E – 0.9 log_10_ C) weight eccentricity and contrast differently. The absolute weight for eccentricity is significantly larger for light stimuli than for dark stimuli (0.76 ± 0.07 vs 0.51 ± 0.06; *p* = 0.031, Bootstrap test), whereas the absolute weight for contrast is significantly larger for dark stimuli than for light stimuli (−1.17 ± 0.09 vs −0.86 ± 0.08; *p* = 0.027, Bootstrap test). Therefore, we conclude that eccentricity affects the visual salience of light stimuli more than dark stimuli, whereas contrast affects the visual salience of dark stimuli more than light stimuli.

### Neuronal mechanisms driving the contrast shift in light/dark visual dominance

**Figure 11.**
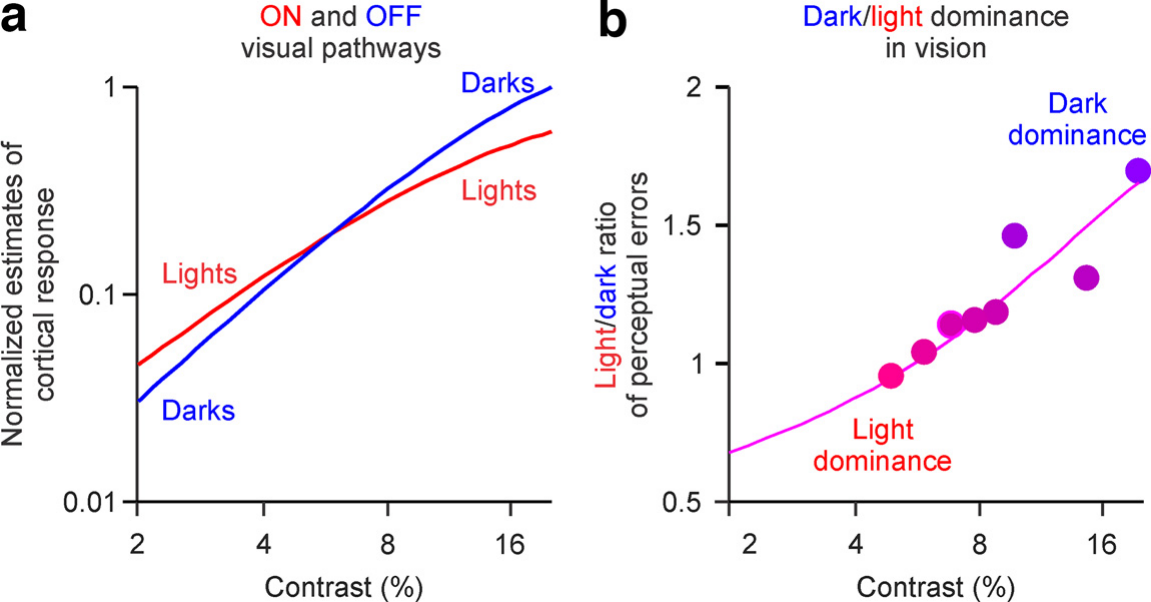
Neuronal mechanism driving the contrast shift in light/dark visual dominance. ***a***, Simulated ON and OFF contrast–response functions with different contrast sensitivity (ON > OFF) and maximum response (OFF > ON) accurately replicate the data from ON-OFF perimetry. Low contrasts drive stronger cortical responses for light than dark stimuli, whereas high contrasts drive stronger cortical responses for dark than light stimuli. ***b***, Light/dark ratios calculated from a division of the contrast response functions shown in ***b*** closely match our measurements in human vision for missed targets averaged across 5° to 30° eccentricities. ON/OFF fitting parameters used to generate Naka–Rushton functions in ***a***: C50, 15%/20%; exponent, 1.5/1.8; R_max_, 1/2. These ON-OFF parameter ratios are similar to those measured in cat visual cortex, cat visual thalamus, human visual cortex, and natural scenes at midgray backgrounds (Kremkow et al., 2014; Rahimi-Nasrabadi et al., 2021).

In many mammalian species, including humans, contrast sensitivity is higher in ON than in OFF retino-thalamo-cortical pathways (Chichilnisky and Kalmar, 2002; Zaghloul et al., 2003; Kremkow et al., 2014; Pons et al., 2017; Ravi et al., 2018; Rahimi-Nasrabadi et al., 2021), whereas the maximum response is stronger in OFF than in ON pathways (Jin et al., 2008; Yeh et al., 2009; Xing et al., 2010, 2014; Kremkow et al., 2014; Zurawel et al., 2014; Wang et al., 2015; Jimenez et al., 2018; Jansen et al., 2019; Oliveira Ferreira de Souza and Casanova, 2019; Rahimi-Nasrabadi et al., 2021). These ON-OFF differences in contrast response functions should have a correlate in visual perception. The higher contrast sensitivity of ON pathways should make light stimuli more visible than dark stimuli at low contrasts, whereas the stronger maximum response of OFF pathways should make dark stimuli more visible than light stimuli at high contrasts (Fig. 11*a*,*b*). Consistently with this prediction, the light/dark ratios measured with ON-OFF perimetry could be accurately replicated by dividing simulated ON and OFF contrast–response functions that have higher ON than OFF contrast sensitivity and stronger OFF than ON maximum responses (Fig. 11*a*). The ON and OFF simulated functions are defined as Rc = R_max_ × C*^n^*^/^(C50*^n^* + C*^n^*), where Rc is the neuronal response to the stimulus contrast, R_max_ is the maximum response, *n* is the exponent, and C50 is the contrast that generates 50% of the maximum response.

In nature, the main parameters of the contrast–response functions (R_max_, C50, and *n*) have larger values in OFF than in ON visual pathways, and these ON-OFF differences are well preserved across species and neuronal structures (Chichilnisky and Kalmar, 2002; Zaghloul et al., 2003; Kremkow et al., 2014; Pons et al., 2017; Rahimi-Nasrabadi et al., 2021). Consistently, in ON-OFF human perimetry, the light/dark ratios of perceptual errors are also best fit by ON/OFF ratios of contrast response functions that have larger R_max_, C50, and *n* values in OFF than in ON functions (Fig. 10*a*,*b*). Also, in cat visual cortex, midgray backgrounds and low luminance ranges make dark–light differences in visual responses more pronounced in R_max_ than in C50 (Kremkow et al., 2014; Pons et al., 2017; Mazade et al., 2019; Rahimi-Nasrabadi et al., 2021). Consistently, in ON-OFF human perimetry, the midgray mean luminance of the background (56 cd/m^2^) and low luminance range of the visual targets (low contrast, ±5 cd/m^2^; high contrast, ±23 cd/m^2^) also made ON-OFF differences larger in R_max_ than in C50 (Fig. 11). Therefore, based on these results, we conclude that the contrast-dependent changes of light/dark dominance in human vision are likely to originate from differences in the contrast response functions between ON and OFF pathways, which are needed to efficiently sample light and dark stimuli in nature (Rahimi-Nasrabadi et al., 2021).

## Discussion

We have demonstrated that luminance contrast affects the visibility of light and dark stimuli differently. At low contrasts, humans locate light targets more accurately and faster than dark targets, but, as contrast increases, dark targets are located more accurately and faster than light targets. This contrast-dependent shift in light/dark dominant polarity can be demonstrated at multiple eccentricities of the human visual field between 5° to 30°, and closely matches the ratio of ON and OFF contrast response functions measured within the retino-thalamo-cortical pathway (Chichilnisky and Kalmar, 2002; Zaghloul et al., 2003; Kremkow et al., 2014; Pons et al., 2017; Ravi et al., 2018; Rahimi-Nasrabadi et al., 2021).

### ON and OFF visual pathways

All animals that can form images on their retinas process light and dark stimuli with separate ON and OFF pathways. ON and OFF pathways respond to opposite light–dark polarities and are differently modulated by the spatiotemporal properties, contrast, luminance, and visual eccentricity of the stimulus (Chichilnisky and Kalmar, 2002; Zaghloul et al., 2003; Jin et al., 2011b; Onat et al., 2011; Komban et al., 2014; Kremkow et al., 2014; Rekauzke et al., 2016; Li et al., 2017; Pons et al., 2017; Luo-Li et al., 2018; Ravi et al., 2018; Jansen et al., 2019; Mazade et al., 2019; Liu et al., 2021; Rahimi-Nasrabadi et al., 2021; Williams et al., 2021). ON and OFF pathways also have a different impact in the visual cortex. Cortical responses to dark stimuli are stronger, faster, more retinotopically precise, and involve a larger number of neurons than cortical responses to light stimuli (Yeh et al., 2009; Xing et al., 2010, 2014; Kremkow et al., 2014, 2016; Zurawel et al., 2014; Lee et al., 2016; Taylor et al., 2018; Jansen et al., 2019). This cortical OFF dominance has been demonstrated in different species including humans (Zemon et al., 1988; Jin et al., 2008; Yeh et al., 2009; Jimenez et al., 2018) and is strongly dependent on stimulus properties such as spatial frequency, size, stimulus duration, luminance range, eccentricity, and contrast (Jin et al., 2008; Onat et al., 2011; Komban et al., 2014; Kremkow et al., 2014; Rekauzke et al., 2016; Jansen et al., 2019; Mazade et al., 2019; Rahimi-Nasrabadi et al., 2021; Williams et al., 2021). Lowering the stimulus contrast should reduce the cortical OFF dominance and make light stimuli more visible than dark stimuli because ON retino-thalamo-cortical pathways have higher contrast sensitivity than OFF cortical pathways (Chichilnisky and Kalmar, 2002; Zaghloul et al., 2003; Kremkow et al., 2014; Pons et al., 2017; Ravi et al., 2018; Rahimi-Nasrabadi et al., 2021). Consistent with this prediction, our results demonstrate that the ratio of light/dark visual dominance is strongly correlated with stimulus contrast. At low contrasts, light stimuli are more visible than dark stimuli, and, as contrast increases, dark stimuli become more visible than light stimuli.

Cortical OFF dominance also decreases with visual eccentricity in both carnivores and rodents (Jin et al., 2008; Williams et al., 2021). In cat visual cortex, OFF thalamic afferents generate stronger current sinks than ON thalamic afferents within the central 5° of the visual field, but the afferent synaptic strength equalizes at more peripheral eccentricities (Jin et al., 2008). Similarly, in mouse visual cortex, dark stimuli drive stronger responses than light stimuli within the central 30° of the visual field (Jimenez et al., 2018; Williams et al., 2021), but, at more peripheral locations, light stimuli drive responses that are equally strong or stronger than those driven by dark stimuli (Smith and Häusser, 2010; Williams et al., 2021). Consistent with these studies, our measurements in humans demonstrate that ON/OFF dominance changes with visual eccentricity. However, unlike the measurements in animal models, the human OFF dominance increases within central vision, mostly from 5° to 15°. Therefore, the OFF dominance in human vision may only decrease at visual eccentricities >30°, which are more similar in spatial resolution to the peripheral vision of carnivores and rodents. In central vision, the human OFF dominance is very robust across different visual tasks, including those that require foveal vision (in Pons et al., 2017: percentage of errors: for darks, 7%; for lights, 15%) and those that require parafoveal and perifoveal vision (percentage of errors at 5−10° of eccentricity with ON-OFF perimetry: for darks, 8%; for lights, 10%).

ON and OFF retinal ganglion cells also sample visual space differently. At the peripheral retina, more cones converge into the larger dendritic fields of ON than OFF retinal ganglion cells (Dacey and Petersen, 1992; Field et al., 2010; Ratliff et al., 2010), and, as a consequence, the receptive fields are larger and retinal ganglion cells are less numerous in ON than in OFF retino-thalamic pathways. These ON–OFF differences in retinal sampling are further amplified in visual cortex, making cortical retinotopy resolution higher for OFF than ON pathways, and spatial resolution higher for dark stimuli than for light stimuli (Chichilnisky and Kalmar, 2002; Kremkow et al., 2014; Pons et al., 2017). These differences between ON and OFF pathways originate early in the retina and are well preserved across the animal kingdom, probably because they are needed to efficiently sample stimuli with different contrast polarity in nature. Light and dark contrasts have different distributions in our visual world, which may explain why contrast sensitivity and contrast saturation are also different in ON compared with OFF pathways (Kremkow et al., 2014; Rahimi-Nasrabadi et al., 2021; Mazade et al., 2022). Light scatter also expands light stimuli and shrinks dark stimuli, which may explain why spatial resolution is lower in ON than in OFF visual pathways (Kremkow et al., 2014; Pons et al., 2017).

Differences in the perception of light and dark stimuli have been recognized by artists, astronomers, philosophers, and psychophysicists for many decades. They were already described by Galileo Galilei (Galilei, 1632), von Helmholtz (von Helmholtz, 1867), Leonardo da Vinci (da Vinci, 1880) and Ernst Mach (Mach, 1886) in past centuries. More recently, psychophysical studies have demonstrated differences between light and dark stimuli in detection threshold, adaptation, visual salience, spatiotemporal resolution, and perception of texture variance and blur (Blackwell, 1946; Short, 1966; Krauskopf, 1980; Whittle, 1986; Poot et al., 1997; Chubb and Nam, 2000; Komban et al., 2011; Lu and Sperling, 2012; Sato et al., 2016; Pons et al., 2017). Analysis of natural scenes also revealed pronounced differences in the distribution of light and dark contrasts in nature (Ratliff et al., 2010; Cooper and Norcia, 2015; Rahimi-Nasrabadi et al., 2021).

### Visual field perimetry

If OFF pathways are faster, have better spatial resolution, and drive stronger cortical responses than ON pathways at most stimulus contrasts, functional deficits in retinal degeneration may be more accurately detected with dark stimuli than light stimuli. Our measurements with ON-OFF perimetry indicate that humans are faster and more accurate at localizing dark than light stimuli when the contrast is >10%. Therefore, although standard visual field perimetry uses light stimuli to detect visual deficits, perimetry with dark stimuli may reduce response variability and increase detection accuracy, which is needed to monitor progression and treatment efficacy in visual diseases such as glaucoma. Glaucoma remains a leading cause of blindness in the world and often affects vision so gradually that visual deficits do not become noticeable to the patient until the disease is advanced. At present, standard visual field perimetry can assess only ON pathway function and requires equipment that is only available at specialized eye clinics. In comparison, the ON-OFF perimetry test that we introduce here measures both ON and OFF pathway function and requires much simpler equipment that may be more accessible to world populations with poor medical resources, small eye clinics, or even private homes (i.e., ON-OFF perimetry requires a headset with eye tracking connected to a computer with a virtual reality-compatible video card).

### Neuronal mechanisms

The strong correlation between light/dark dominance and stimulus contrast that we demonstrate can be explained by differences in the contrast response functions of ON and OFF cortical pathways. ON thalamo-cortical pathways have higher contrast sensitivity than OFF pathways, and the difference is strongly dependent on background luminance and luminance range (Kremkow et al., 2014; Pons et al., 2017; Rahimi-Nasrabadi et al., 2021). The low luminance range of ON-OFF perimetry should drive much stronger cortical responses to dark stimuli than light stimuli. Consistent with this interpretation, the correlations that we demonstrate between contrast and light/dark ratios of visual dominance are accurately simulated with OFF pathways that generate stronger responses but have lower contrast sensitivity than ON pathways. In summary, our results provide a link between the physiology of ON and OFF cortical pathways and human visual perception that could have potential applications to measurements of visual field perimetry and contrast sensitivity in the clinical setting.
